# Identification of differences in microRNA transcriptomes between porcine oxidative and glycolytic skeletal muscles

**DOI:** 10.1186/1471-2199-14-7

**Published:** 2013-02-18

**Authors:** Yingkai Liu, Mingzhou Li, Jideng Ma, Jie Zhang, Chaowei Zhou, Tao Wang, Xiaolian Gao, Xuewei Li

**Affiliations:** 1 Institute of Animal Genetics & Breeding, College of Animal Science & Technology, Sichuan Agricultural University, Ya’an, Sichuan, China; 2 Department of Biology & Biochemistry, University of Houston, Houston, TX, USA

**Keywords:** microRNA, Deep sequencing, *Longissimus doris* muscle, *Psoas* major muscle, Pig

## Abstract

**Background:**

MicroRNAs (miRNAs) are a type of non-coding small RNA ~22 nucleotides in length that regulate the expression of protein coding genes at the post-transcriptional level. Glycolytic and oxidative myofibers, the two main types of skeletal muscles, play important roles in metabolic health as well as in meat quality and production in the pig industry. Previous expression profile studies of different skeletal muscle types have focused on these aspects of mRNA and proteins; nonetheless, an explanation of the miRNA transcriptome differences between these two distinct muscles types is long overdue.

**Results:**

Herein, we present a comprehensive analysis of miRNA expression profiling between the porcine *longissimus doris* muscle (LDM) and *psoas* major muscle (PMM) using a deep sequencing approach. We generated a total of 16.62 M (LDM) and 18.46 M (PMM) counts, which produced 15.22 M and 17.52 M mappable sequences, respectively, and identified 114 conserved miRNAs and 89 novel miRNA*s. Of 668 unique miRNAs, 349 (52.25%) were co-expressed, of which 173 showed significant differences (*P* < 0.01) between the two muscle types. Muscle-specific miR-1-3p showed high expression levels in both libraries (LDM, 32.01%; PMM, 20.15%), and miRNAs that potentially affect metabolic pathways (such as the miR-133 and -23) showed significant differences between the two libraries, indicating that the two skeletal muscle types shared mainly muscle-specific miRNAs but expressed at distinct levels according to their metabolic needs. In addition, an analysis of the Gene Ontology (GO) terms and KEGG pathway associated with the predicted target genes of the differentially expressed miRNAs revealed that the target protein coding genes of highly expressed miRNAs are mainly involved in skeletal muscle structural development, regeneration, cell cycle progression, and the regulation of cell motility.

**Conclusion:**

Our study indicates that miRNAs play essential roles in the phenotypic variations observed in different muscle fiber types.

## Background

Skeletal muscle is a type of highly heterogeneous tissue that is traditionally divided into red (type I and IIa) and white (type IIb) fiber types [1]. Red skeletal muscles (such as the *psoas* major muscle) can undertake chronic contractile activity without fatigue because they are better endowed with capillaries, myoglobin, lipids, and mitochondria than are white muscles (such as the *longissimus doris* muscle) [2]. To improve understanding of the major factors that determine the phenotypic properties of red and white muscles, previous studies have been performed at the mRNA level. Campbell *et al*. identified 49 differentially expressed mRNA sequences using a microarray approach [3]. Bai *et al*. developed a porcine skeletal muscle cDNA microarray and revealed numerous candidate genes involved in muscle phenotype determination [4]. Recently, transcriptional analysis between the red and white skeletal muscle of Chinese Meishan pigs revealed 28 signaling pathways including insulin signaling and a cell cycle pathway that responded to metabolic differences between muscle types [5]. Studies at the protein level have also revealed dissimilarities between the white and red skeletal muscle. White muscle was composed predominantly of glycolytic enzymes, whereas red muscle had a greater abundance of contractile proteins with higher oxidative enzyme content [6,7]. Gelfi *et al*. used two-dimensional gel electrophoresis and mass spectrometry to build a reference map of proteins and identified hundreds of distinct gene products including metabolic, transport, and contractile proteins [8]. Moreover, it was reported that the different energetic demands of white and red muscles were matched primarily by the different numbers of mitochondrial proteins in the two tissues [9]. Recently, a number of studies have focused on small non-coding RNAs, such as microRNAs (miRNAs) and small interfering RNAs (siRNAs), which are involved in the transcriptional and post-transcriptional regulation of gene expression. Nonetheless, how these small RNAs are poised to perform different tasks in different muscle types has rarely been described.

miRNAs are ubiquitously expressed non-coding small RNAs of ~22 nt in length. They are encoded by genes in the nucleus where miRNA primary precursors (pri-miRNAs) are formed. After processing with the Drosha and Dicer RNases, stem-loop precursor miRNAs (pre-miRNAs) are transcribed into mature miRNAs [10]. The 2–8 nucleotides at the 5′ end of a miRNA are termed the functional ‘seed’ region for the recognition of target mRNAs. The miRNA is loaded into an RNA-induced silencing complex, which decreases translation of the targeted gene product [11,12]. miRNAs have myriad roles in muscle biology and many are expressed in a tissue- and/or stage-specific manner [13]. In 2006, miR-1 and miR-206 were reported to regulate the *myostatin* gene which directly impacts muscular hypertrophy [14]. Increasing evidence has shown that the miRNAs can regulate the expression of transcription factors and signaling mediators for myopathies and muscular dystrophies [15-17]. Muscle-specific miR-1 and -133 are transcriptionally regulated by myogenic differentiation factors (e.g., *MyoD*, *Mef2*, and *SRF*) [18], deletion of these miRNAs resulted in aberrant muscle maintenance. A cluster of miRNA species (miR-23, -103, -107, and -278) were proposed to affect metabolic pathways by fine-tuning gene expression patterns [19]. Furthermore, miRNAs also play important roles in myogenic differentiation [18] and development [20-22]. Nonetheless, little is known about the differentially expressed miRNAs in white and red skeletal muscles.

Pigs are of significant agricultural value and are considered an ideal model system for biomedical research [23]. The application of deep sequencing has greatly accelerated the discovery of porcine miRNAs [24-26]. Understanding differentially expressed miRNAs in different muscle tissues will facilitate further identification of functional miRNA biomarkers, which also are potential candidates for further improvement of meat quality and production using molecular approaches.

Here, we studied the distinct porcine miRNA expression patterns between the representative *longissimus doris* (white) and *psoas* major (red) muscles and investigated the roles of miRNAs in regulating transcriptome networks involved in the two types of muscle fiber. Our results extend the repertoire and understanding of porcine skeletal muscle miRNAs.

## Results and discussion

### Phenotypic differences between the two distinct muscles

**Figure 1:**
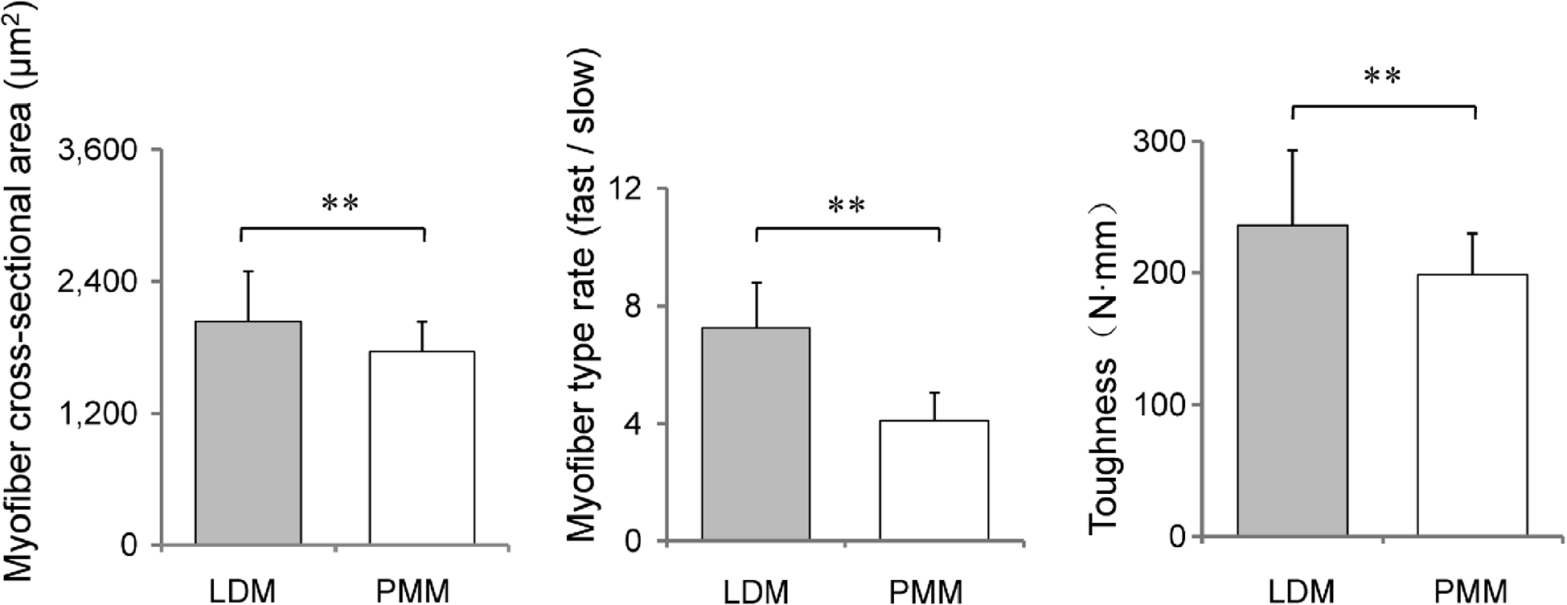
**Phenotypic differences between the two distinct muscles.** LDM, *longissimus dorsi* muscle; PMM, *psoas* major muscle. Statistical significance was calculated by one-way repeated-measures ANOVA (*n* = 3). ** *P* < 0.001.

Compared with the *psoas* major muscle (PMM), the *longissimus doris* muscle (LDM) exhibited a higher cross-sectional area, myofiber type rate, and toughness (Student’s *t*-test, *P* < 0.001) (Figure 1), which suggests that there may be a disparity in the molecular mechanisms behind these differences.

### Summary of deep sequencing data

The sequencing of two small RNA libraries from the LDM and PMM yielded 16.62 M and 18.46 M counts of sequenced sequences (sequ-seqs), respectively (Additional file 1: Figure S1A). Of which, 15.22 M and 17.52 M LDM and PMM sequences, respectively, accounting for > 90% of the total sequ-seqs, were considered mappable sequences after filtering out the sequ-seqs that did not meet the accepted criteria. The statistics of the distribution of small RNAs while applying a series of filters are given in Additional file 2: Table S1. The reads (LDM: 0.69 M, 4.14%; PMM: 0.51 M, 2.74%) that mapped to certain other known classes of RNA sequences (i.e. mRNA, rRNA, tRNA, snRNA, snoRNA, and repetitive sequence elements) were eliminated from the analysis (Additional file 1: Figure S1B). The proportions of sequ-seqs that mapped to the other RNA classes are listed in Additional file 3: Table S2. There was a positive correlation between the expression level of the total counts of all isomiRs and the most abundant sequence (LDM, Pearson’s *r* = 0.98; PMM, Pearson’s *r* = 0.97), thus, we used the most abundant sequence (Additional file 1: Figure S1C) and its count to represent a family of sequences that varies by length and/or by one nucleotide as previously report [20].

The size distribution of mappable sequences was similar in both libraries (Pearson’s *r* = 0.99). The most abundant size class among the small RNA sequences was the 22 nt RNAs, accounting for 9.12 M (LDM, 59.91% of all mappable sequences) and 10.87 M (PMM, 62.07% of all mappable sequences), followed by the 21 and 23 nt RNAs (Additional file 1: Figure S1D). This distribution is consistent with the typical 21–23 nt range for miRNAs from Dicer-derived products [27]. Notably, almost all of the other RNAs in the LDM library had higher expression levels than the equivalent RNAs in the PMM library, with the exception of the 19 nt RNAs.

### Muscle-specific miRNAs

As shown in Table 1, we divided the mappable reads into three groups (Additional file 4: Table S3) in order from high-to-mid confidence [28]: (1) known porcine, 305 miRNAs corresponding to 282 known porcine pre-miRNAs that were also mapped to the pig genome. Specifically, 171 miRNAs and 50 miRNA*s are known in miRBase, 86 have not been identified and are novel (Additional file 5: Table S4.1); (2) novel porcine, 117 miRNAs corresponding to 114 other known miRBase mammalian (pig not included) pre-miRNAs that were mapped to the pig genome (Additional file 5: Tables S4.2), this is in agreement with previous observations that many miRNAs are conserved among species [29]; and (3) candidate porcine, 381 miRNAs (longer than 18 nt and unmapped to any known miRBase mammalian pre-miRNAs) encompassing 355 candidate pre-miRNAs, with predicted RNA hairpins derived from the pig genome (Additional file 5: Tables S4.3).

**Table 1:** Known porcine and conserved miRNAs detected in LDM and PMM

**Group (number of miRNA/pre-miRNA)**	**LDM**	**PMM**
Known porcine miRNAs	252/273	278/300
(also mapped to genome)		
Novel porcine miRNAs	74/77	83/85
(also mapped to genome)		
Candidate porcine miRNAs	100/107	223/235

miRBase 17.0 (April, 2011) documented 228 pre-miRNAs encoding 257 known porcine miRNAs. In this study, we found 204 and 218 known porcine miRNAs in LDM and PMM, respectively, indicating that these two libraries encompass almost all of the known porcine miRNAs. Only 31 of the known miRNAs were undetected in our data sets, likely because of extremely low or no expression in these two skeletal muscles.

Notably, 97 of the 204 (LDM), and 108 of the 218 (PMM) known porcine miRNAs, produced multiple mature variants from the reported miRNAs in miRBase, probably because of the existence of isomiRs expressed over a range of levels [30,31]. It has been suggested that the most abundant isomiRs may vary across tissues or developmental stages [32]. Here, the most frequently observed isomiR was chosen as a reference sequence. In addition, 68 (LDM) and 84 (PMM) corresponding miRNA*s were detected for the first time in this study. In most cases, the novel miRNA*s were typically low in abundance when compared with the known miRNAs, perhaps explaining why they have not been detected previously. Low abundance miRNAs frequently exhibit rapid turnover for biological regulation, and the detection of these novel miRNAs demonstrates the high sensitivity of the deep sequencing method [33,34].

After the mappable reads were mapped to known porcine and/or other conserved mammalian miRNAs, the remaining unmapped mappable reads were designated candidate porcine miRNAs. Based on a series of filters (see Methods), 100 (LDM) and 223 (PMM) miRNAs corresponding to 107 and 235 pre-miRNAs were predicted and the corresponding miRNA*s of 12 (LDM) and 24 (PMM) were identified. Intriguingly, about a quarter of these novel candidates mapped to two or more loci in the genome (Additional file 5: Tables S4.3), this may be related to time- and space-specific expressions as described previously [35].

### Differentially expressed miRNAs between LDM and PMM

**Figure 2:**
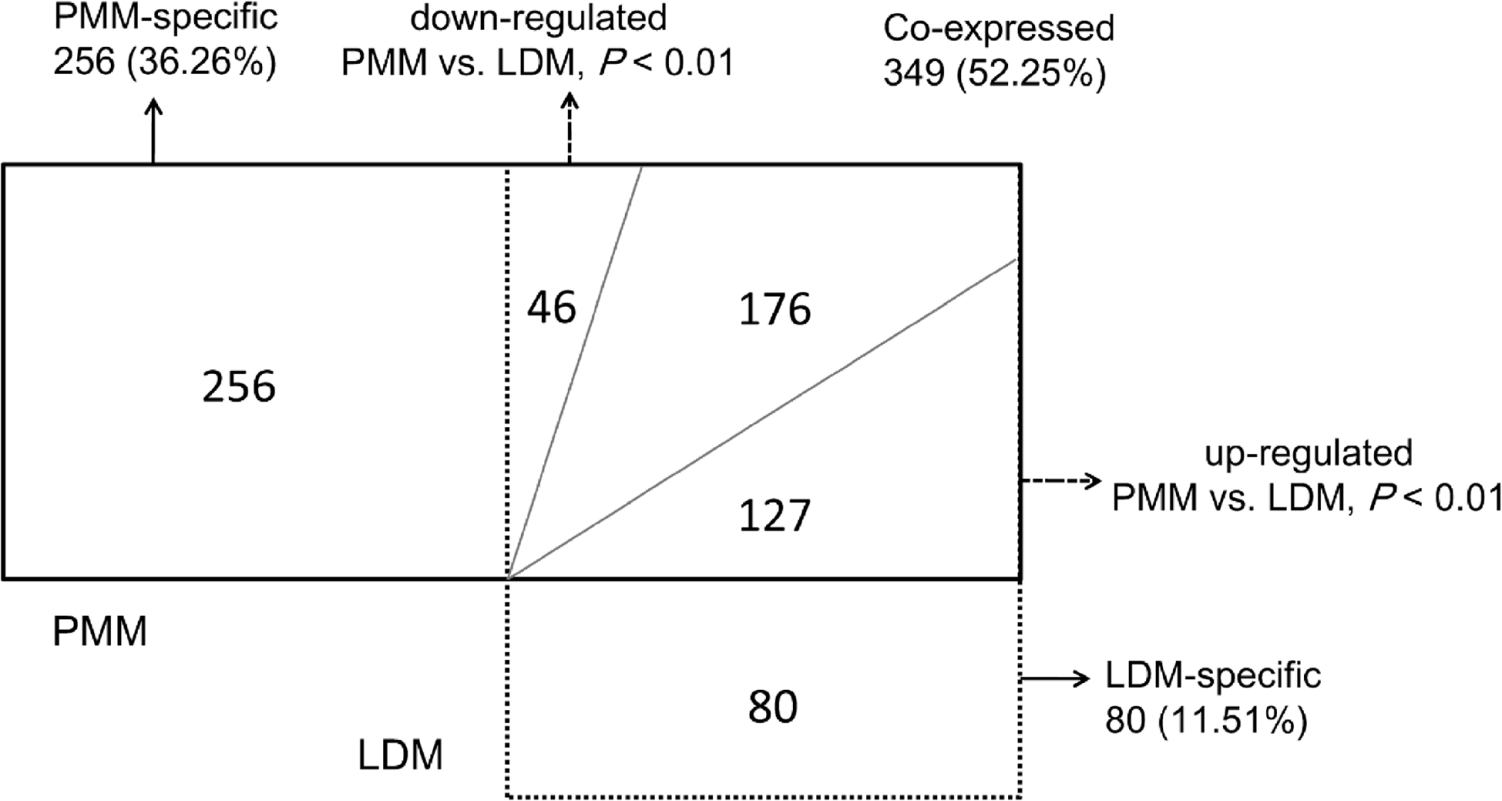
**Characteristics of differentially expressed miRNAs between LDM and PMM.** The diagram displays the distribution of 668 unique miRNAs between the PMM (solid line) and LDM (dash line) libraries. The dashed arrows indicate the differentially expressed unique miRNAs (*P* < 0.001) in PMM against LDM. The solid arrows indicate PMM- and LDM-specific miRNAs. LDM, *longissimus dorsi* muscle; PMM, *psoas* major muscle.

We found that 349 of 668 (52.25%) unique miRNAs were co-expressed in the two libraries, and 80 and 256 miRNAs were specifically expressed in the LDM and PMM libraries, respectively, possible reflecting the physiological differences between these two distinct muscle types. Since the tissue-specific miRNAs accounted for less than 0.02% of the total counts, we concentrated on the differentially expressed, relatively abundant miRNAs in each library. To determine the significance of differences in the miRNA counts in each library, the IDEG6 program [36] was used for the normalization calculation between the mappable sequences in the two libraries. A unique miRNA was considered to be differentially expressed when a Fisher’s exact test produced *P* < 0.001 [37]. By applying this criterion, we identified 173 miRNAs (out of 349 co-expressed) that were differentially expressed between the PMM and LDM libraries (Figure 2, Additional file 6: Table S5), including 127 and 46 up- and down-regulated miRNAs across the two libraries.

**Figure 3:**
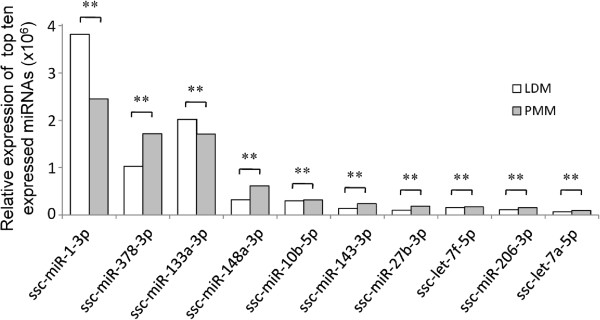
**Top ten unique miRNAs with the highest expression levels in LDM and PMM.** The top ten unique miRNAs were co-expressed in the PMM and LDM libraries. The IDEG6 program was used for the normalization calculation and all ten miRNAs showed differential expression between the two libraries (**P < 0.001, Fisher’s exact test). LDM, *longissimus dorsi* muscle; PMM, *psoas* major muscle.

The known miRNAs exhibited a very broad range of expression that varied from three to several hundreds of thousands of sequence reads, only a few the miRNAs dominated the miRNA abundances. As shown in Figure 3, the top ten unique miRNAs with the highest expression levels accounted for 92.46% (LDM) and 88.82% (PMM) of the total unique miRNA counts. Notably, miR-1 and -133a, the highly-characteristic muscle-specific miRNAs, represented more than half of the total copy number for PMM and LDM, respectively. Their very high abundance reflects the important regulatory role that they play in skeletal muscle proliferation and differentiation [18,38,39], the genes containing the potential target sites are likely to be highly expressed in muscle tissue. miR-10b has also been observed in relatively high abundance in skeletal muscles [25] and, although little is known about its functional role in myoblast growth, an analysis of its target genes demonstrated that it was involved in myogenesis regulation and participates in muscle development regulation [38].

All of the top ten unique miRNAs were co-expressed in both the PMM and LDM libraries but with different ranks. Our results are consistent with previous studies that found that miR-1, -133, and -206 frequently ranked among the highest expressed miRNAs in porcine muscle cell proliferation and differentiation [25,40,41]. The let-7a and -7f miRNAs were ubiquitously expressed in both tissues and accounted for over 1.90% of the total reads. Four of the top ten miRNAs, miR-378, -148a, -143, and -27b, were up-regulated (more than 1.5-fold change, *P* < 0.001) in PMM compared with LDM; however, there have been no reports documenting their possible *in vivo*/*vitro* roles in muscle development. Therefore, we carried out a prediction analysis for the target genes of these four differentially expressed miRNAs using three prediction programs, PicTar [42], TargetScan [43], and miRNA-Target Gene Prediction at EMBL [44]. A total of 888 target genes corresponding to 440 unique mRNA-miRNA interactions were predicted by combing the results from the three programs. The subsequent DAVID gene annotation analysis [45] of the predicted target genes indicated that the most enriched GO terms included transcription regulation, macromolecule biosynthetic processes, cellular protein metabolic processes, muscle cell differentiation, and skeletal muscle development (Additional file 7: Table S6). According to the KEGG pathway annotation of their putative target protein coding genes (Additional file 8: Table S7), focal adhesion and the MAPK, Wnt, and mTOR signaling pathways were identified as the global changes between porcine red (PMM) and white (LDM) muscles. Interestingly, the enriched ECM-receptor interaction is reportedly associated with meat tenderness and texture [46]. Our data also highlighted pathways related to cancer, indicating that the genes expressed in cell proliferation and differentiation were targeted by other miRNAs. In summary, the annotations for the predicted targets indicate that different metabolic patterns are regulated by miRNAs between porcine PMM and LDM muscles. Further investigation is required to better understand the influence of miRNAs on the phenotypes of the various muscle fiber types.

Our results demonstrate that differential metabolic mechanisms may drive the development of the two muscle types in the direction of their individual functions. We identified several miRNAs that participate in muscle metabolic pathways and showed that these exhibit significant differences between the PMM and LCM libraries (Additional file 9: Figure S2). Both miR-1 and -181 promote mammalian myoblast differentiation and development [47], and it has been speculated that miR-1 plays a role in inducing antioxidant response in skeletal muscle [48]. Our finding that miR-1 accounted for 32.01% of the detected miRNAs in our LDM library is consistent with previous results. Others have reported that miR-133a enhances myocyte proliferation by reducing protein levels of SRF and inhibits polypyrimidine tract-binding protein (nPTB) translation; both of these proteins are crucial regulators for muscle differentiation [49,50]. miR-181 was barely detectable in resting muscle cells [47] and miR-206 was only highly expressed in newly formed muscle fibers [51]. This may explain why they were present in relatively low abundance in adult skeletal muscles compared with the high abundance of the muscle-specific miR-1 and miR-133 in this study. The PMM and LDM libraries showed similar expression levels of miR-103 and miR-107, both of which have been predicted in the pathways that involve cellular Acetyl-CoA and lipid levels [46]. The *PGC1α* gene is known to be a reliable target of miR-23 [48], which increases the mitochondrial content of mouse skeletal muscle [52]. A previous study has shown that exercise-induced *PGC1α* usually led to the generation of more oxidative red muscle than oxidative white muscle fibers [53]. In our study, both the sequencing and microarray profiling results revealed that the LDM library possessed a slightly higher abundance of miR-23 than the PMM library (~1.75-fold), which might lead to relatively lower expression of *PGC1α* in LDM. The differences between the two muscle types are mainly determined by the relative ratio of muscle fibers, miR-23 and its target *PGC1α* are just one of the complex components of the metabolic mechanisms that are involved. In summary, we tentatively conclude that the differentially expressed miRNAs between the two libraries contribute in a major way to the development of skeletal muscles in the respective directions required for them to carry out their functions.

### Microarray validation

To further validate the deep sequencing results, microarrays to investigate the relative expression levels of the miRNAs in both libraries were performed. First, the three biological replicates were highly correlated with each other (average Pearson’s *r* = 0.95, *P* < 10^-16^, Table 2 and Additional file 10: Figure S4), suggesting experimental reliability and making it possible to pool the samples during the sequencing process in case of individual differences. Second, we correlated the microarray and deep sequencing results. As a result, 436 unique miRNAs including 151 known porcine miRNAs were detected and 110 of 436 miRNAs were the same as the sequ-seqs. For the known miRNAs, the Pearson’s correlation between the microarray profiling and sequencing results was 0.67 (Additional file 11: Figure S3, *P* < 0.001). The top ten expressed miRNAs also showed strong signals in the microarrays, with the exception of miR-206, Pearson’s correlation was 0.83 (*P* < 0.001). Microarray profiling identified miR-26a as being highly expressed in both libraries, however, this highly expressed miRNA was not identified by sequencing. These differences may derive from the intrinsic differences between these two approaches [54-56].

**Table 2:** Pearson’s correlation of the counts of 1623 unique miRNAs among three biological replicates in two libraries (**: *P* **< 10**^**-16**^**)**

**Sample No. (LDM/PMM)**	**2**	**3**
1	0.95^**^/0.95^**^	0.98^**^/0.88^**^
2		0.96^**^/0.97^**^

## Conclusions

Our study has indicated the essential roles that miRNAs play in different muscle fiber types and should make studies of the muscle-specific regulation involving miRNAs possible.

## Methods

### Animal ethics statement

All research involving animals was conducted according to the Regulations for the Administration of Affairs Concerning Experimental Animals (Ministry of Science and Technology, China, revised in June 2004) and approved by the Institutional Animal Care and Use Committee in the College of Animal Science and Technology, Sichuan Agricultural University, Sichuan, China under permit No. DKY-B20121406.

### Small RNA library construction and sequencing

The *longissimus doris* (LDM) and *psoas* major muscles (PMM) were obtained from three female Landrace pigs (210-days-old), immediately frozen in liquid nitrogen, and then stored at -80°C. The mirVana™ miRNA isolation kit (Ambion, Austin, TX, USA) was used to extract small RNA following the manufacturer’s procedure. Total RNA were tested for quality and purity using a NanoDrop ND-1000 spectrophotometer (Nano Drop, DE, USA) at 260/280 nm (ratio > 2.0). The integrity of total RNA was also monitored via analysis by the Bioanalyzer 2100 and RNA 6000 Nano LabChip Kit (Agilent, CA, USA) with RIN number > 6.0. Qualified RNA was prepared for sequencing samples as follows: equal quantities (5 *μ*g) of total RNA isolated from the individual females were pooled. Approximately 45 *μ*g of total RNA, from each tissue was used for both library preparation and sequencing. The 10–40 nt short RNAs were isolated by polyacrylamide gel electrophoresis (PAGE) and combined with proprietary adaptors (Illumina, San Diego, CA, USA). The small RNA fractions were then converted to cDNA by RT-PCR and the cDNA was sequenced on the Genome Analyzer GA-2 (Illumina) following the manufacturer’s recommended protocol for small RNA sequencing.

### Identification and profiling of differentially expressed miRNAs between LDM and PMM

Sequenced sequences (Sequ-seqs) were modified as per our previous reports [20] after processing with Illumina’s Genome Analyzer Pipeline software. Mappable sequences were generated after applying a series of additional filters [34,37]. The mappable sequences were then mapped to the pig genome (~2.26 Bbp, Sscrofa9: http://asia.ensembl.org/Sus_scrofa/Info/Index) in three steps using the NCBI local BLAST package (http://blast.ncbi.nlm.nih.gov/): (1) the 228 known porcine pre-miRNAs (encoding 257 miRNAs) and 5804 known pre-miRNAs (encoding 6271 miRNAs) from 22 other mammals in miRBase 17.0 (http://www.mirbase.org/) were mapped to the genome; (2) the mappable sequ-seqs were mapped to the pig genome to obtain their genomic locations and the corresponding annotations from Ensembl release 59 (Sscrofa9, April 2009); and (3) UNAFold [24] was used to predict the hairpin RNA structures of the mappable sequences not-mapped to miRBase in step 1 from the adjacent 60 nt sequences in either direction. To avoid ambiguous sequ-seqs that were assigned to multiple positions in the pig genome, only sequ-seqs longer than 18 nt were used for further analysis in step 3.

### Microarray for validation

To confirm the deep sequencing results, six customized miRNA microarrays corresponding to 1572 pig-specific miRNA probes were used to evaluate the expression of porcine miRNAs. Microarray probes were collapsed to miRNAs by taking the median expression values of the respective probes per miRNA. Extracted log_2_-transformed intensities were quartile normalized to make all data comparable.

## Competing interests

The authors declare that they have no competing interests.

## Authors’ contributions

YKL carried out the molecular genetic studies, participated in the sequence alignment, and drafted the manuscript. MZL participated in the study design and revised the manuscript. TW and JDM carried out the microarrays and data analysis. XLG participated in the sequence alignment. JZ and CWZ collected the samples and performed the statistical analysis. XWL conceived of the study and participated in its design and coordination and helped to draft the manuscript. All authors read and approved the final manuscript.

## Supplementary Material

Additional file 1: Figure S1 Summary of the sequencing results. **(A)** Distribution of the sequencing data in each library after applying a series of filters. LDM, *longissimus dorsi* muscle; PMM, *psoas* major muscle. **(B)** Distribution for the other known classes of RNA sequences. **(C)** Mappable reads were divided into three groups. **(D)** Length distribution and frequency percentage of the unique miRNAs. The Y-axis indicates the ratio of miRNA (numbers of each stage divided by total numbers in a library). LDM, *longissimus dorsi* muscle; PMM, *psoas* major muscle. Click here for file

Additional file 2: Table S1 Statistics of the distribution for small RNAs during a series of filters in order. Click here for file

Additional file 3: Table S2 Proportion of sequ-seqs mapping to known RNA classes. Click here for file

Additional file 4: Table S3 Statistics based on the counts of the mappable sequences. Click here for file

Additional file 5: Table S4 Profile of the known porcine miRNAs (miRBase 17.0) with genome locations. **Table S4.2.** Profile of the known other mammalian miRNAs (miRBase 17.0). **Table S4.3.** Profile of porcine candidate miRNAs. Click here for file

Additional file 6: Table S5 Unique porcine miRNAs. Click here for file

Additional file 7: Table S6 List of the top 20 enriched Gene Ontology (GO) terms. Click here for file

Additional file 8: Table S7 KEGG pathways enriched for the predicted protein coding target genes of miR-378, -148a, -143, and -27b. Click here for file

Additional file 9: Figure S2 Relative expression levels of metabolism-related miRNAs. LDM, *longissimus dorsi* muscle; PMM, *psoas* major muscle. **P <0.001, Fisher’s exact test. Click here for file

Additional file 10: Figure S4 Correlation of miRNA expression among three biological replicates within two libraries. **(A)** A scatter plot and trend line (Pearson’s correlation) revealed a correlation between the log10 of miRNA expression of each biological replicate. Line represents linear regression. **(B)** Heat map matrix of Pearson’s correlation between individuals and tissues. Click here for file

Additional file 11: Figure S3 Deep sequencing and microarray data correlations. The data shows the fold change of relative expression between *longissimus dorsi* muscle (LDM) and *psoas* major muscle (PMM). **(A)** Correlation of the 151 known porcine miRNAs. **(B)** Correlation of the top ten co-expressed miRNAs. Click here for file
