# Supplementary figures and images for: Identification of differences in microRNA transcriptomes between porcine oxidative and glycolytic skeletal muscles
Source: BMC Mol Biol. 2013 Feb 18;14:7. doi: 10.1186/1471-2199-14-7 (PMC3599761; doi:10.1186/1471-2199-14-7)

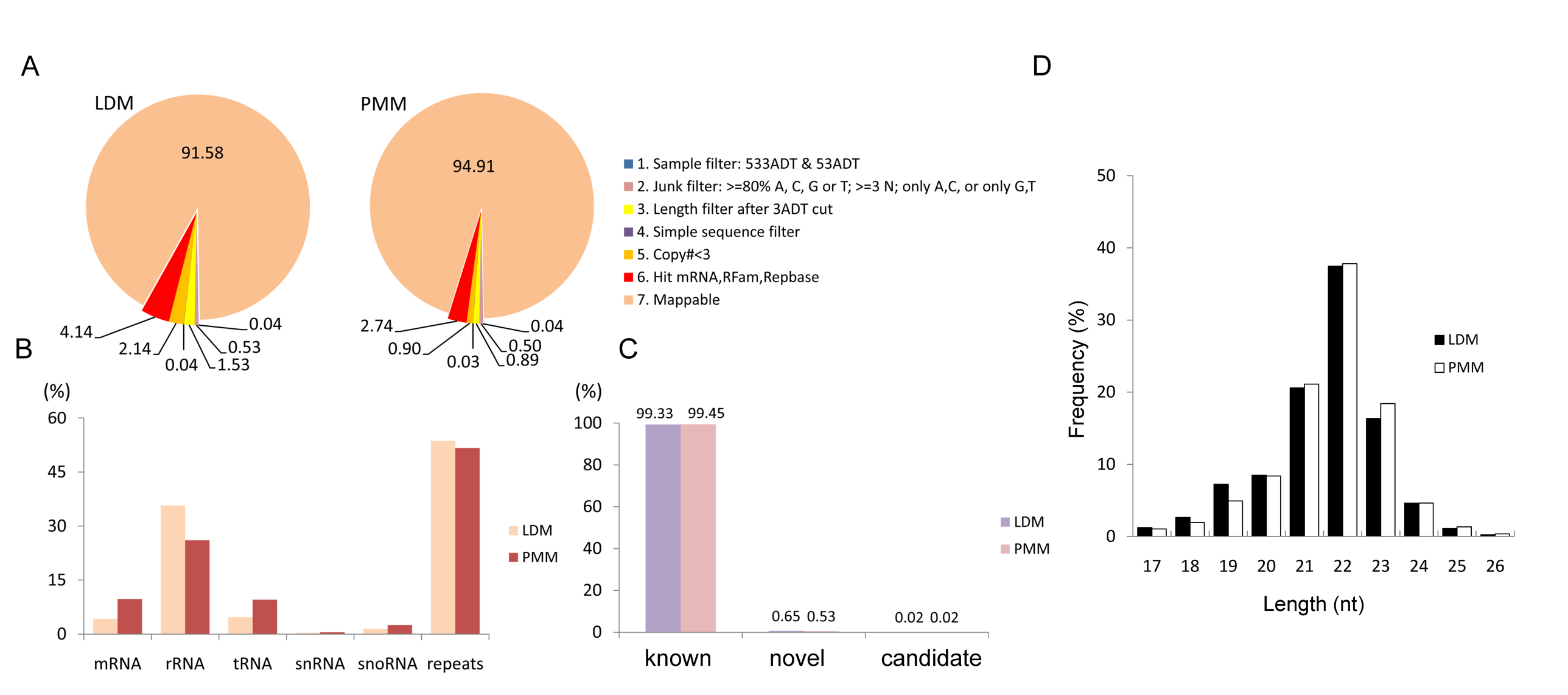

Supplement: Additional file 1: Figure S1 — Summary of the sequencing results. (A) Distribution of the sequencing data in each library after applying a series of filters. LDM, longissimus dorsi muscle; PMM, psoas major muscle. (B) Distribution for the other known classes of RNA sequences. (C) Mappable reads were divided into three groups. (D) Length distribution and frequency percentage of the unique miRNAs. The Y-axis indicates the ratio of miRNA (numbers of each stage divided by total numbers in a library). LDM, longissimus dorsi muscle; PMM, psoas major muscle. [file 1471-2199-14-7-S1.tiff]

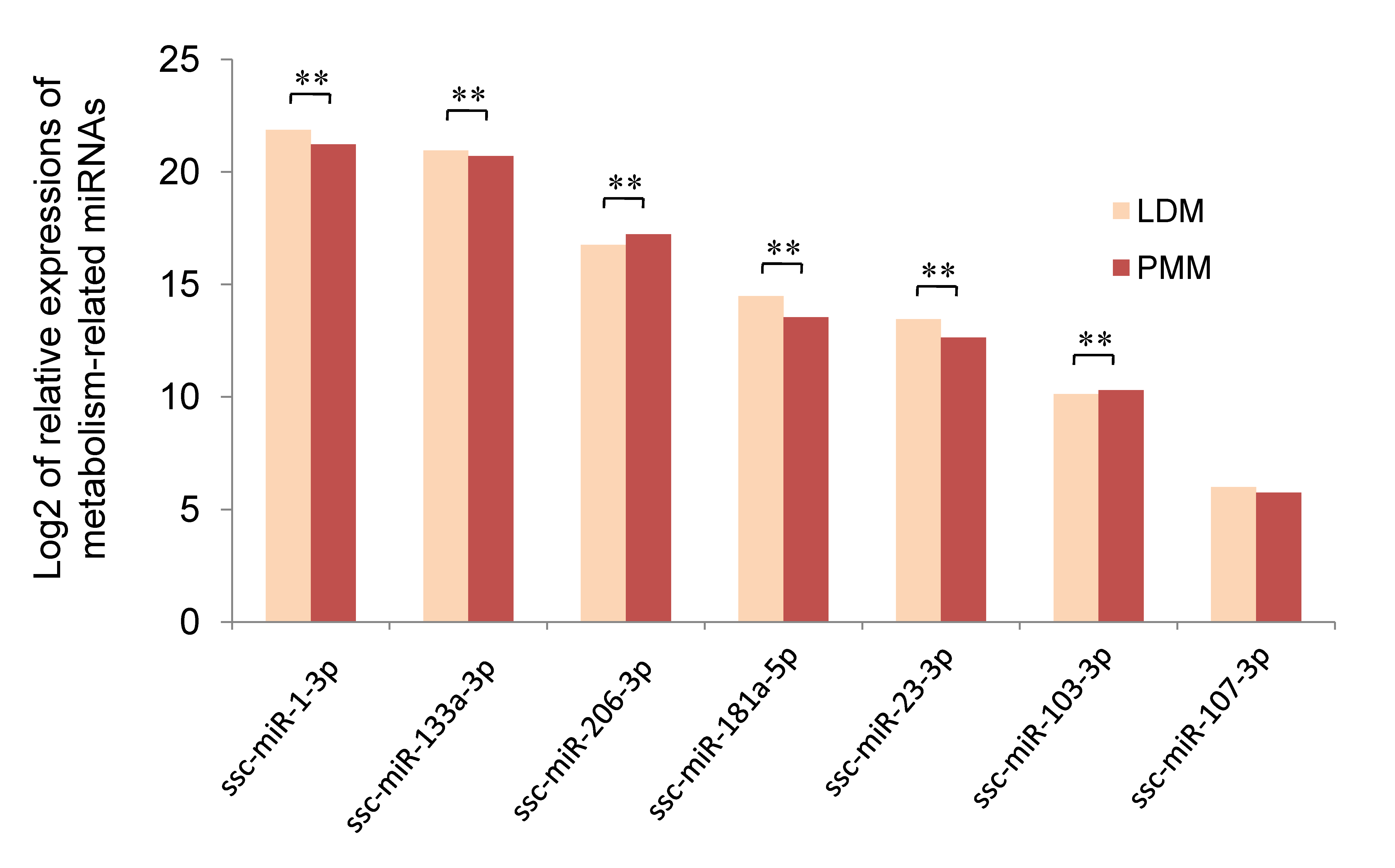

Supplement: Additional file 9: Figure S2 — Relative expression levels of metabolism-related miRNAs. LDM, longissimus dorsi muscle; PMM, psoas major muscle. **P <0.001, Fisher’s exact test. [file 1471-2199-14-7-S9.tiff]

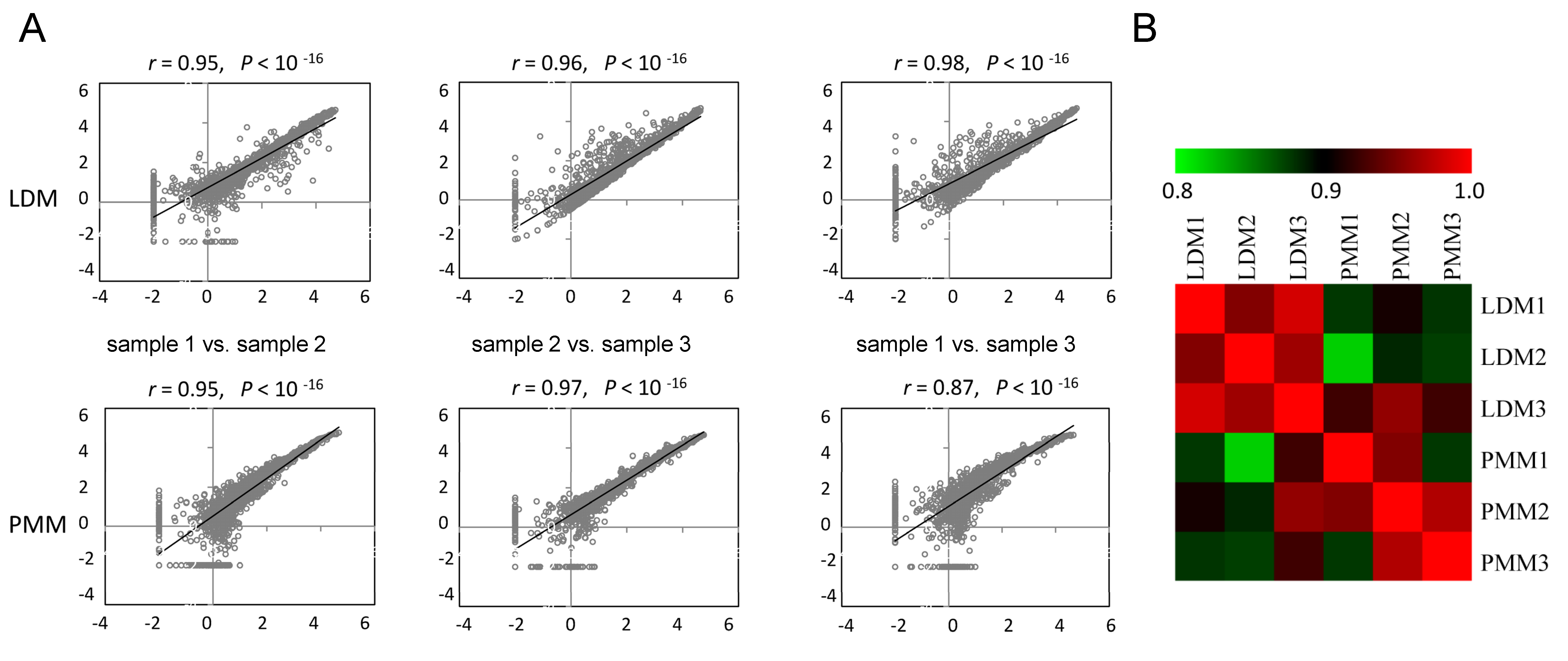

Supplement: Additional file 10: Figure S4 — Correlation of miRNA expression among three biological replicates within two libraries. (A) A scatter plot and trend line (Pearson’s correlation) revealed a correlation between the log10 of miRNA expression of each biological replicate. Line represents linear regression. (B) Heat map matrix of Pearson’s correlation between individuals and tissues. [file 1471-2199-14-7-S10.tiff]

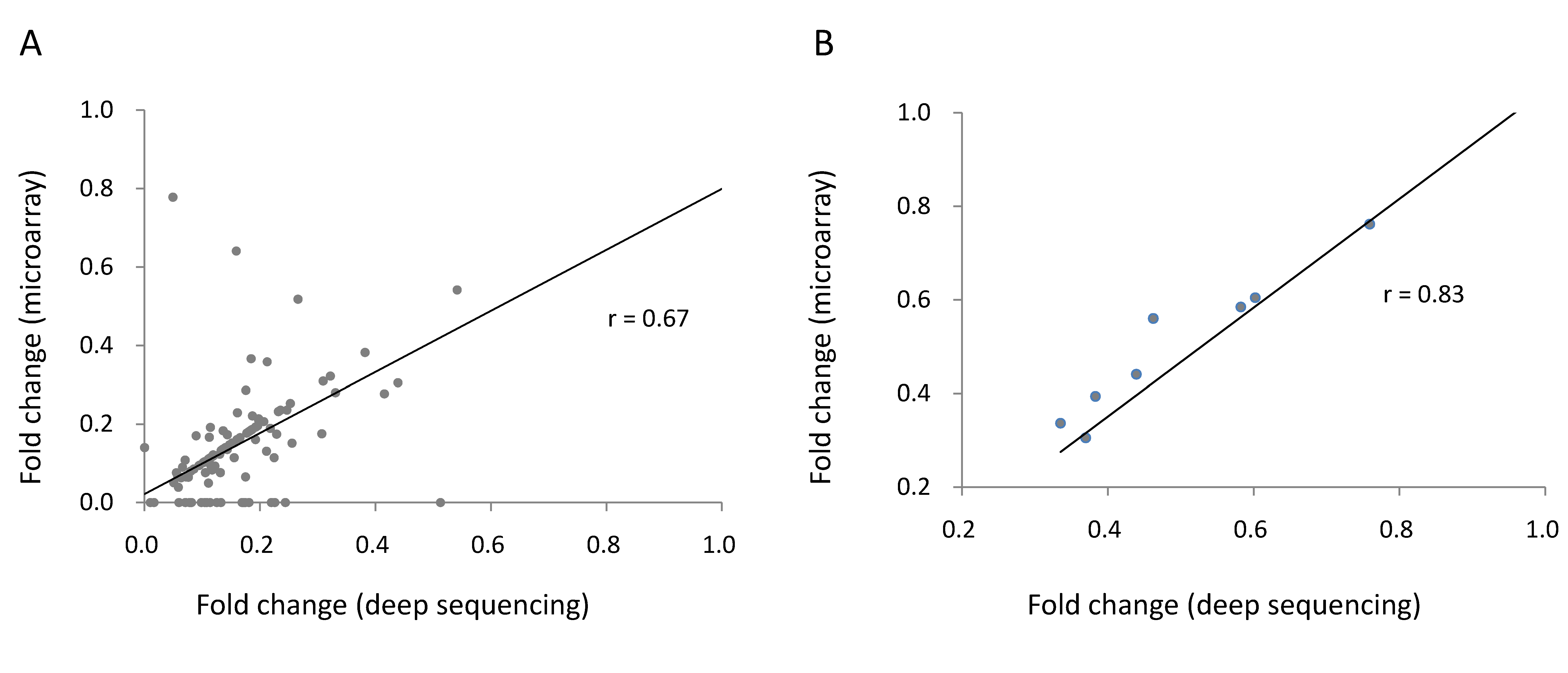

Supplement: Additional file 11: Figure S3 — Deep sequencing and microarray data correlations. The data shows the fold change of relative expression between longissimus dorsi muscle (LDM) and psoas major muscle (PMM). (A) Correlation of the 151 known porcine miRNAs. (B) Correlation of the top ten co-expressed miRNAs. [file 1471-2199-14-7-S11.tiff]
